# Preeclampsia and gestational weight gain in the Norwegian Fit for Delivery trial

**DOI:** 10.1186/s13104-018-3396-4

**Published:** 2018-05-08

**Authors:** E. R. Hillesund, S. Seland, E. Bere, L. R. Sagedal, M. K. Torstveit, H. Lohne-Seiler, I. Vistad, N. C. Øverby

**Affiliations:** 1 Department of Public Health, Sport and Nutrition, University of Agder, Serviceboks 422, 4604 Kristiansand, Norway; 2 Department of Obstetrics and Gynecology, Sørlandet Hospital HF, Serviceboks 416, 4604 Kristiansand, Norway; 3 Department of Research, Sørlandet Hospital HF, Serviceboks 416, 4604 Kristiansand, Norway

**Keywords:** Preeclampsia, Primipara, Gestational weight gain, Body composition, Fat mass

## Abstract

**Objective:**

Excessive gestational weight gain is linked to risk of preeclampsia, but it is not clear whether the association is causal. The purpose of this paper was to examine gestational weight gain in the Norwegian Fit for Delivery study among women who developed preeclampsia compared to those who did not, and to further explore associations between weight gain and preeclampsia by including data on body composition (bioimpedance) assessed in the last trimester of pregnancy.

**Results:**

A total of 550 women were eligible for the study. Women who developed preeclampsia gained more weight than women who did not (difference 3.7 kg, p = 0.004), with a 3.5 kg difference in total body water observed in week 36 (p = 0.040). Adjusted for age, education, pre-pregnancy body mass index (BMI), randomization, and fat mass, a one kg increase in GWG was associated with 1.3 times higher odds of preeclampsia (OR: 1.31, 95% CI 1.15–1.49, p < 0.001). An independent inverse association between fat mass in week 36 and odds of preeclampsia was observed (OR: 0.79, 95% CI 0.68–0.92, p = 0.002). Given the observed difference in total body water, these findings point to excess fluid as the component driving the association between gestational weight gain and preeclampsia in the present study.

*Trial registration* The NFFD trial has the Clinical Trials registration: clinicaltrial.gov NCT0100168

## Introduction

Preeclampsia is a serious complication of pregnancy with an incidence rate of 2–8% worldwide [1]. Preeclampsia is defined as the development of hypertension and proteinuria after 20 weeks of gestation [2] and is a leading cause of maternal mortality and morbidity, perinatal deaths, and preterm delivery [3]. Preeclampsia is also associated with intrauterine growth restriction with immediate and long-term risks for the child [4]. The causes of preeclampsia are not well established. A leading hypothesis is placental dysfunction from early pregnancy [5] that leads to placental ischemia and an exaggerated maternal systemic inflammatory reaction to pregnancy. Maternal gestational weight gain (GWG) has received attention as a potentially modifiable risk factor for preeclampsia, however the results are inconclusive, with some studies suggesting an association [6–9] and others not [10, 11]. The fact that gestational weight gain (GWG) is a composite of fat mass, fat-free mass, and extracellular fluid accrual complicates the interpretation of these observations.

The aim of this paper is to examine gestational weight gain (GWG) of women in the Norwegian Fit for Delivery trial (NFFD) who developed preeclampsia compared to those who did not, and to further explore associations between GWG and preeclampsia by including body composition data measured in the last trimester of pregnancy.

## Main text

### Methods

Data were derived from the Norwegian Fit for Delivery study, a randomized controlled trial where 606 nulliparous women were included and randomized to a lifestyle intervention group (physical activity sessions and dietary guidance) or a control group (clinicaltrial.gov NCT0100168) [12]. Participants were recruited by midwives at eight health care clinics in the Southern part of Norway between September 2009 and February 2013. Inclusion criteria were primiparity, age above 18 years, body mass index (BMI) above 19, and being literate in either Norwegian or English. Exclusion criteria were twin gestation, pre-existing diabetes, and inability to participate in the physical activity part of the program. Participants had to be recruited into the study before pregnancy week 20.

The current paper used data from all participants regardless of randomization assignment. In total 550 women of the 606 participants originally randomized were included in the present analysis.

#### Subjects

Of the 606 women, 32 were excluded due to not meeting inclusion criteria (19 with BMI < 19 kg/m^2^, five recruited too late in pregnancy, 3 abortions, 3 moving away, one not nulliparous, and one pregnant with twins). In total 19 participants withdrew their consent to participate after inclusion and randomization. A total of 13 of these did not give a reason, while 4 said they did not want to participate in the sports activity, one did not want to be in the control group, and one did not want to take blood tests. In addition, five more were excluded because of missing information on preeclampsia.

#### Instruments

Pre-pregnancy weight was based on self-reported values given in kilograms (kg). Participants were also weighed at the health care clinic at study inclusion on scales that were calibrated at study start. Every participant had two separate examinations at Sørlandet hospital (gestational week 30 and 36) where weight was measured with a bio-impedance scale (Tanita BC 418, Tokyo, Japan) with 0.1 kg precision. The scale measures fat percentage, total fat mass, total fat-free mass and total body water. The women were measured with light clothing and no shoes. Weight at delivery was measured by a SECA weight with 0.1 kg precision. If weight at delivery was not recorded, the last weight measured at the health care clinic were used. Height was measured with a portable stadiometer (Seca Leicester, Hamburg, Germany) at the consultation in week 30. Pre-pregnancy BMI in kg/m^2^ was calculated using self-reported weight and height measured in week 30. Weight gain in pregnancy was calculated both from self-reported pre-pregnancy weight and from weight measured at study inclusion. Total pregnancy weight gain was only calculated for women who gave birth after week 37. Weekly weight gain in each trimester was computed for all participants. Excessive GWG was defined as > 16, > 11.5 and > 9 kg if normal weight, overweight and obese pre-pregnancy, respectively, according to Institute of Medicine (IOM) recommendations [13].

Participants responded to a questionnaire at inclusion and in week 36. Descriptive information on age at inclusion, smoking, randomization assignment, and education were collected from the baseline questionnaire. Educational attainment was categorized as ≤ 12, 13–15, and ≥ 16 years.

Preeclampsia was diagnosed based on guidelines adopted by the Norwegian Federation of Obstetricians and Gynecologists; an increase in blood pressure to at least ≥ 140 mm Hg systolic or 90 mm Hg diastolic after 20th gestational week combined with proteinuria (protein excretion of at least 0.3 g/24 h or ≥ 1+ on dip-stick), both measured at least twice [14]. Severe preeclampsia was defined as preeclampsia before 34 weeks of pregnancy and/or severity of symptoms, as documented in hospital charts, including cases of eclampsia and HELLP (hemolysis, elevated liver enzymes, low platelet count). All cases were ascertained retrospectively from hospital charts.

#### Statistical analysis

Independent sample t-tests were performed for normally distributed data, mainly those related to weight development. Values are given in mean and standard deviation (SD). Mann–Whitney U-test was used to compare non-normally distributed data and are presented as median and interquartile range (Q1–Q3). Chi square tests were performed for comparison of categorical data. We assessed associations between total GWG (continuous in kg) and excessive GWG (yes/no) and preeclampsia in crude and multivariate binary logistic models that were adjusted for maternal age, education, randomization assignment, pre-pregnant BMI, and fat mass in week 30 or 36, respectively. Associations are presented as odds ratios (OR) with 95% confidence intervals (CI). A p-value of < 0.05 was considered significant.

### Results

Of the 550 women included, 25 (4.5%) developed preeclampsia, with a non-significant difference of 3.6% in the intervention group and 5.4% in the control group. Among the 25 preeclampsia cases, 15 (60%) were classified as severe.

There was no difference in maternal age, gestational age at inclusion, educational attainment, smoking habits, or diabetes prevalence between those with and without preeclampsia (Table 1). Those who developed preeclampsia had significantly shorter pregnancy duration than those who did not (p < 0.001), and more often instrumental delivery (p < 0.001) because of induced delivery and planned cesarean section.

**Table 1:** Description of participants, including the whole sample, participants with no preeclampsia and participants with preeclampsia

	Whole sample (n = 550)	No preeclampsia (n = 525)	Preeclampsia (n = 25)	p-value^a,b,c^
Age, years	28.0 (4.4)	28.0 (4.3)	29.4 (4.6)	0.116
Gestational age at inclusion	108 (17)	108 (17)	106 (20)	0.607
Belonging to intervention group, %	49.8	50.3	40.0	0.424
Smoking, %	3.8	3.8	4.0	1.0
Gestational diabetes, diet-regulated, %	8.4	8.4	8.0	0.862
Gestational diabetes, insulin, %	1.1	1.1	0.0	
Pregnancy duration, weeks	40 (39–41)	40 (39–41)	38 (36–39)	< 0.001
% instrumental delivery	21.5	19.8	60.9	< 0.001
Education				
3-year high school or less	30.8	30.8	32.0	0.523
University/university college less than 4 years	33.8	34.2	24.0	
University/university college more than 4 years	35.4	35.0	44.0	

^a^For normally distributed data, Independent-samples t-test was used with values presented as mean (SD). ^b^ For non-normally distributed data, Mann–Whitney U-test was used and values given as median (Q1–Q3). ^c^ For categorical variables, Chi square test was performed, and values given in percent

There were no significant differences regarding maternal pre-pregnancy weight, maternal or paternal pre-pregnancy BMI, maternal fat mass, or % body fat between those who developed preeclampsia and those who did not, but participants who developed preeclampsia had higher total body water in week 36 (difference 3.5 kg, p = 0.040) (Table 2).

Participants who developed preeclampsia had higher total GWG (difference 3.7 kg, p = 0.004) and higher weekly weight gain as calculated from pre-pregnancy to study inclusion (difference 88 g/week, p = 0.031), from inclusion to week 30 (difference 131 g/week, p = 0.005), and from week 30 to 36 (difference 210 g/week, p = 0.004) (Table 2).

**Table 2:** Comparison of weight and weight development between women without and with preeclampsia

	Total sample (n = 550)	No pre-eclampsia (n = 525)	Preeclampsia (n = 25)	Difference	p-value^a,b^
Pre-pregnancy weight (kg)^a^	68.0 (12.1)	68.0 (12.0)	67.5 (14.6)	0.5	0.840
Pre-pregnancy BMI (kg/m^2^)^a^	23.8 (3.8)	23.8 (3.8)	24.2 (4.0)	− 0.3	0.657
Normal-weight, %^b^	71.6	71.2	80.0		0.604
Overweight, %^b^	21.5	20.8	16.0		
Obese, %^b^	7.8	8.0	4.0		
Paternal BMI, (kg/m^2^)^a^	25.5 (3.2)	25.6 (3.2)	24.9 (3.5)	0.6	0.377
Weight gain in pregnancy, kg^a^ (from pre-pregnancy weight)	15.1 (6.1)	14.9 (6.0)	18.6 (7.1)	− 3.7	0.004
Weight gain in pregnancy, kg (from weight at inclusion)^a^	12.7 (5.0)	12.6 (4.9)	14.8 (6.0)	− 2.2	0.040*
Weight gain per week from pre-pregnancy to inclusion, grams^a^	152 (196)	148 (194)	236 (217)	− 88	0.031*
Weight gain per week from inclusion to week 30, grams^a^	504 (214)	498 (210)	629 (279)	− 131	0.005*
Weight gain per week from week 30–36, grams^a^	554 (298)	547 (293)	757 (361)	− 210	0.004*
Fat percentage, week 30, %^a^	35.4 (5.2)	35.4 (5.2)	35.0 (5.8)	0.4	0.732
Fat mass, week 30, kg^a^	28.0 (8.5)	28.0 (8.4)	29.2 (11.6)	− 1.2	0.523
Fat percentage. week 36, %^a^	35.5 (5.2)	35.5 (5.2)	34.6 (6.8)	1.0	0.483
Fat mass, week 36, kg^a^	29.3 (8.8)	29.2 (8.6)	31.6 (14.6)	− 2.4	0.537
Total body water, week 30, kg^a^	36.4 (3.6)	36.3 (3.5)	38.0 (5.7)	− 1.7	0.188
Total body water, week 36, %^a^	37.7 (3.9)	37.8 (3.8)	41.2 (5.9)	− 3.5	0.040*
Excessive GWG (%)^b^	51.7	47.2	73.9	− 26.7	0.012*

Weight gain in pregnancy (based on pre-pregnancy weight): missing = 21. Weight gain in pregnancy (based on weight at study inclusion): missing = 26. Weight gain per week from pre-pregnancy to inclusion: missing = 18

Weight gain per week from inclusion to week 30: missing = 35. Weight gain from week 30 to 36: missing = 45. Fat percentage and fat mass in week 30: missing = 51. Total Body Water in week 30: missing = 49. Total Body Water in week 36: Missing = 58

Excessive GWG according to Institute of Medicine 2009 [13], a total of 536 women

^a^Independent-samples t-test. ^b^ Chi square test. * Significance level p < 0.05

Adjusted for maternal age, education, pre-pregnancy BMI, randomization, and fat mass in week 30, a 1 kg increase in total GWG increased the odds of preeclampsia (OR: 1.21, 95% CI 1.09–1.34, p < 0.001). The association was similar when fat mass in week 30 was replaced by fat mass in week 36 in the model (OR: 1.31, 95% CI 1.15–1.49, p < 0.001). An independent *inverse* relationship between fat mass and odds of preeclampsia was observed in both models (OR: 0.85, 95% CI 0.74–0.98, p = 0.021 for fat mass measured in week 30, and OR: 0.79, 95% CI 0.68–0.92, p = 0.002 for fat mass measured in week 36). An independent association between pre-pregnancy BMI and preeclampsia was also observed (OR: 1.43, 95% CI 1.08–1.90, p = 0.012) adjusted for fat mass in week 30, and OR: 1.71, 95% CI 1.26–2.31, p = 0.001 adjusted for fat mass in week 36).

We reran the adjusted models with *excessive* GWG as the main exposure. Excessive GWG was associated with higher odds of preeclampsia (OR: 3.54, 95% CI 1.15–10.91, p = 0.028 adjusted for fat mass in week 30, and OR: 5.43, 95% CI 1.34–21.98, p = 0.018 adjusted for fat mass in week 36). Neither fat mass nor any other covariates were independently associated with preeclampsia in these models.

To assess potential influence of the lifestyle intervention we reran the adjusted models confined to the control group. All associations remained significant in the restricted sample (data not shown).

### Discussion

Women in the NFFD study who developed preeclampsia gained significantly more weight throughout pregnancy than women who did not, and 3 in 4 with preeclampsia had excessive GWG according to IOM recommendations. A significant association between GWG and preeclampsia remained after adjustment for age, education, pre-pregnancy BMI, randomization, and 3rd trimester fat mass. The association was not driven by increased fat mass as evidenced by the fact that fat mass was inversely associated with preeclampsia risk.

The difference in gestational weight gain among women with and without preeclampsia was evident early, with significant higher weight gain throughout pregnancy in women who developed preeclampsia. There was no difference between the groups in fat mass in week 30 and 36, but an increasing difference in total body water during the same time window, suggesting edemas at that point in preeclamptic pregnancies. The difference in total gestational weight gain almost equaled the difference in total body water in week 36.

Our findings regarding associations between GWG and preeclampsia are in line with several other studies [6, 7, 9], although there is still conflicting evidence [10, 11]. Underlying mechanisms for a potential causal association between weight gain per se and preeclampsia could be that excessive GWG may increase oxidative stress, and thereby stimulate or aggravate a systemic inflammatory response which could accelerate damage to vascular endothelial cells leading to preeclampsia [15]. Magnus et al. argue that reverse causation must be considered as an explanation of the association between GWG and preeclampsia because edemas will cause increased weight gain [16]. Our findings by including body composition measurements in the models support this view.

To date, lifestyle interventions targeting GWG through diet and physical activity have not been convincingly successful in reducing the prevalence of preeclampsia, even with successful reductions in GWG [17]. If excessive GWG is a consequence of preeclampsia rather than a causal factor in the etiology of preeclampsia it may be prudent to focus more on diet quality and other health behaviors that may modify the stress response to pregnancy rather than on modifying weight gain per se.

Weight gain was larger in all three trimesters in preeclamptic pregnancies, even in early pregnancy when edemas are not likely to be present. To our knowledge this has not been reported previously. This raises the question whether early rapid (or excessive) weight gain could be indicative of underlying pathophysiological processes and should lead to closer attention and intensified follow-up. Tiralongo et al. found differences in maternal hemodynamics in the first trimester of pregnancy that could influence risk preeclampsia [18]. Of note, pre-pregnancy weight was self-reported, so the comparison of weight gain before inclusion is less reliable and should not be given too much weight.

In conclusion, the present study supports the previously described association between gestational weight gain and preeclampsia. The association was, however, not driven by increased fat mass. We therefore question whether excessive GWG is a causal factor in the pathophysiology of preeclampsia, or rather an indication of early endothelial dysfunction leading to excess fluid retention from an early stage of pregnancy. Early weight gain combined with measurement of body composition in early pregnancy should be further explored in relation to subsequent development of preeclampsia.

## Limitations

Strengths to this study are the weight and height measurements performed with calibrated scales at several time points throughout pregnancy, and the body composition measurements performed in gestational weeks 30 and 36 of pregnancy. Pre-pregnancy weight was self-reported, thus differences in weight gain in early pregnancy is less reliable than in later pregnancy. Løf et al. assessed body composition at several timepoints throughout pregnancy and concluded that bioimpedance spectroscopy is potentially useful although increases in TBW during pregnancy tends to be underestimated [19].

The limited number of preeclampsia cases and the numerical difference between cases and non-cases might compromise confidence in the magnitude of the effect estimates in the present analyses. The small sample of cases, and the fact that standard deviations were larger for all body weight-related variables in the preeclampsia group, would tend to reduce the likelihood of identifying true differences between the groups.

Residual and unmeasured confounding may remain, but we repeated all analyses confined to the control group alone to address potential residual confounding due to the lifestyle intervention. Due to similar findings, we chose to present the results from the total sample with adjustment for randomization assignment. Generalizations should, however, be done with caution as women were recruited from a limited geographical area in Norway and represents a relatively highly educated population.

## Abbreviations

BMI: body mass index
CI: confidence interval
GWG: gestational weight gain
HELLP: hemolysis, elevated liver enzymes, low platelet count
IOM: Institute of Medicine
NFFD: Norwegian Fit for Delivery study
OR: odds ratio
SD: standard deviation
